# Confidence of Faculty in Writing Letters of Recommendation for Pediatric Fellowship Applicants

**DOI:** 10.7759/cureus.49750

**Published:** 2023-11-30

**Authors:** Christopher J Moran, Kristina Dzara, Ariel S Frey-Vogel, Michael Flaherty, Daniel Hall, Benjamin A Nelson, Katherine Sparger, Takara Stanley, Phoebe Yager, Shannon Scott-Vernaglia

**Affiliations:** 1 Division of Pediatric Gastroenterology, Hepatology, and Nutrition, Mass General for Children, Boston, USA; 2 Department of Pediatrics, Harvard Medical School, Boston, USA; 3 Department of Family and Community Medicine, Saint Louis University School of Medicine, Saint Louis, USA; 4 Center for Educator Development, Advancement, and Research, Saint Louis University School of Medicine, Saint Louis, USA; 5 Division of General Pediatrics, Mass General for Children, Boston, USA; 6 Division of Pediatric Critical Care Medicine, Mass General for Children, Boston, USA; 7 Division of Pediatric Pulmonology, Mass General for Children, Boston, USA; 8 Division of Neonatology, Mass General for Children, Boston, USA; 9 Division of Pediatric Endocrinology, Mass General for Children, Boston, USA

**Keywords:** medical education, faculty development, gender bias, pediatric fellowship, letters of recommendation

## Abstract

Background: The assessment of pediatric residents applying to subspecialty fellowship programs relies on faculty letters of recommendation (LOR). However, it is unclear if pediatric faculty are confident that their LOR are effective.

Objective: This study aims to assess the confidence of pediatric faculty in writing an effective LOR for pediatric residents applying to subspecialty fellowship programs.

Methods: Survey development was conducted using evidence-based best practices. Surveys were distributed via email in 2021 to all full-time pediatric faculty members who taught pediatric residents in a large academic medical center. Categorical values were compared by chi-square test.

Results: Eighty-five out of 150 (57%) faculty members completed the survey. Forty-one percent of participants were very confident that their LOR provided adequate content to assess residents during the application process. Confidence was associated with higher academic rank (p=0.02), frequent contact with residents (p=0.01), and writing >2 LOR in the last five years (p=0.0002). Confident LOR writers were more likely to describe their own background, details about the resident’s scholarly activity, and the resident’s ability to work as part of a team. Thirty-five percent of respondents reported never considering gender bias when writing LOR, whereas 28% reported always considering gender bias. Eighty-seven percent of respondents reported an interest in receiving LOR writing guidelines.

Conclusion: Half of the faculty respondents were not very confident in their ability to write an effective LOR for pediatric residents applying for a fellowship. Faculty development and standardized instructions on writing effective LOR may be helpful both at the institutional and national levels, including the importance of considering gender bias when writing LOR.

## Introduction

Applying to a subspecialty fellowship training program is a complex process for pediatric residents. Letters of recommendation (LOR) are one of the most important factors considered by fellowship program directors (PDs) when reviewing applications [1]. LOR are often solicited by trainees from faculty with whom they have worked closely, although it is not clear how confident faculty are at writing LOR. Fellowship PDs reviewing LOR will frequently look for specific code phrases to indicate the strength of the recommendation [2]. The LOR review process is complicated by assessment inflation with less experienced LOR writers more likely to give higher ratings [3-5].

A full complement of LOR for an application to training programs includes both a primary LOR (from a clerkship director for medical students applying to residency programs or from a PD for residents applying to fellowship programs) and multiple secondary LOR. The style of LOR written by clerkship directors to residency programs has strongly trended toward standardized LOR (sLOR) [6-8]. More recently, guidelines from the Alliance for Academic Internal Medicine (AAIM) have advocated for internal medicine residency PDs to transition to a sLOR for fellowship applicants [9]. These AAIM guidelines have resulted in improved trust in the LOR written by residency PDs for trainees applying for internal medicine fellowships [10]. Pediatric otorhinolaryngology has implemented similar strategies for the PD LOR [11].

Most residency and fellowship programs require a secondary LOR written by faculty who have worked closely with the applicant. These LOR are written by faculty who may have had specific clinical or research experiences with the applicant and often take a narrative approach. There are minimal guidelines on what to include in narrative LOR for applications to residency programs across medical subspecialties (with even less guidance for LOR to fellowship programs), and specific words chosen by an LOR writer to describe an applicant can be interpreted by PDs differently than their intended meaning [12-15]. Complicating the art of writing LOR is that criteria for pediatric fellowship selection differ from those for residency selection, as fellowship PDs report a stronger focus on prior participation in scholarly work, publications, and presentations at academic conferences compared to residency PDs [2]. Although an instrument has been developed for writing effective LOR for pediatric residency applicants, this tool may not apply to writing LOR for fellowship applicants given that pediatric fellowship PDs report distinct priorities in application review such as participation in research [2,16].

Adding to the complexity of writing LOR is the potential impact that implicit bias has on the content and strength of LOR. Applicant gender heavily impacts word choice and topics included in LOR for many subspecialties including general surgery and radiology [5,17-19]. Gold et al. demonstrated that gender bias influences clinical evaluations for pediatric residents with male pediatric residents being more likely to be cited for intellect and preparedness and female residents being more likely to be cited for enthusiasm and caring [20]. In addition, pediatric LOR have recently been shown to have a bias toward describing leadership and ambition [21]. More concerning, extensive literature describes the effect of gender and racial bias on the perceived strength of the LOR [5,19]. A subset of LOR writers may not even be aware of the potential for this type of bias, which may impact how they refer to the trainee, both in descriptive words and titles used.

While there are data demonstrating variability in LOR among faculty, no data are describing the confidence levels of faculty (in any medical specialty) in writing these LOR. This study assessed the confidence of full-time pediatric faculty members in a large academic medical center in their ability to write an effective LOR for pediatric residents applying to subspecialty fellowship programs and explored preparation and practices related to writing LOR. A secondary goal was to identify the habits of confident LOR writers to inform recommendations for LOR writing.

## Materials and methods

The survey tool was designed utilizing evidence-based best practices in survey design that were informed by Artino et al. [22]. The specific focus of this survey was on LOR writing for pediatric residents applying to subspecialty fellowship training programs. The survey was designed to assess common preparatory activities for writing the LOR as well as the likelihood of the inclusion of factors determined to be integral based on the literature review. We performed a literature search to identify similar surveys with validity evidence and synthesized these with additional survey items as deemed necessary by those study team members who were frequent letter writers for residents applying for pediatric fellowship. Once the survey was finalized, cognitive interviewing was conducted to ensure the clarity of the survey questions. The survey was then pilot-tested by three pediatric subspecialists at other similarly-sized pediatric departments in academic medical centers, and the final implementation of the survey was done with the target audience.

Faculty demographics included academic rank (professor, associate professor, assistant professor, and instructor), the number of LOR written by the participant in the last five years, and how often the participant worked with residents (more than once per week, once per week, less than once per week). Participants were asked to categorize their confidence in writing LOR as very confident, somewhat confident, or not confident. Participants were surveyed on preparatory items that they requested prior to writing an LOR, specific topics that they included in the LOR, and whether they considered gender bias while writing the LOR. Participants were also asked if they had ever declined a request to write an LOR for a resident. Full details are available in the Appendices section.

Surveys were designed in REDCap (REDCap, Tennessee, USA) and distributed via email once in 2021 to all full-time faculty members with clinical responsibilities as well as all affiliated continuity clinic preceptors within the pediatric faculty of one large academic center (n=150), Mass General for Children, Boston, USA. Participants could skip any question if desired. Mass General Brigham Human Research Protection Committee approved the study.

Statistical analyses were performed using the chi-square test for survey results in GraphPad Prism 9.3.0 (GraphPad Software, California, USA) comparing the three levels of confidence (very confident, somewhat confident, and not confident) across variables. The statistical significance threshold was set at a p-value of <0.05.

## Results

Eighty-five participants completed the survey among 150 eligible faculty (57% response rate). Survey respondent academic rank included 5% professor (N=4/82), 16% associate professor (N=13/82), 41% assistant professor (N=34/82), and 35% instructor (N=29/82). Twenty percent of participants held current residency or fellowship PD or associate/assistant PD roles (Table 1). Declining a request to write an LOR for a resident was reported by 6.1% of participants.

**Table 1:** Respondent details LOR: letter of recommendation

Medical school rank	% (n)
Professor	5% (4)
Associate professor	16% (13)
Assistant professor	41% (34)
Instructor	35% (29)
Other	2% (2)
Active leadership role in a training program	20% (16)
Number of LOR written in the last 5 years	
None	26% (22)
1-2	35% (29)
>2	39% (33)
Frequency of working with residents	
Less than a weekly basis	43.9% (36)
Weekly basis	29.3% (24)
More than once per week basis	26.8% (22)

**Figure 1:**
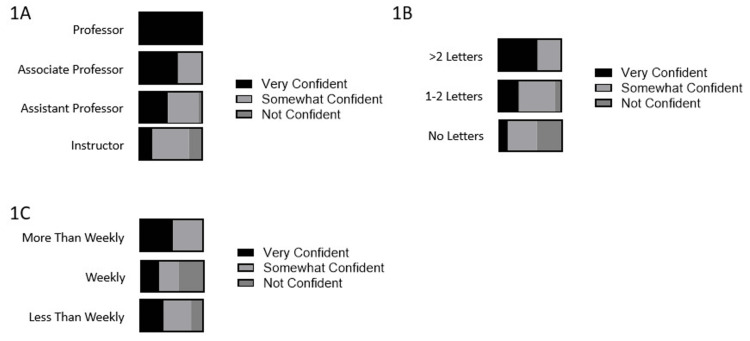
Confidence levels of pediatric faculty

Overall, 41% of participants reported feeling very confident that their LOR provided fellowship programs with the information needed to effectively evaluate applicants. Higher confidence levels were associated with higher faculty medical school rank (p=0.02), a greater number of letters written in the last 5 years (p=0.0002), and the frequency with which the participant worked with residents (p=0.01) (Figure 1). Having a current leadership role in a pediatric training program was not associated with confidence in LOR writing (p=0.40).

The most commonly requested preparatory pieces of information for writing an LOR were an updated curriculum vitae (86%) and a one-on-one meeting with the resident (45%). There were no specific preparatory steps associated with LOR writing confidence (Table 2).

**Table 2:** Preparatory requests prior to writing an LOR (% reporting always performing a task) stratified by confidence in LOR writing LOR: letter of recommendation

	Very confident	Somewhat confident	Not confident	p-value
One-on-one meeting	42%	43%	50%	0.47
Updated curriculum vitae	90%	83%	83%	0.69
Drafted personal statement	42%	47%	33%	0.85
LOR drafted by the applicant	6%	0%	0%	0.60
Prioritized topics to include	29%	11%	33%	0.23

The most common LOR features included describing how the participant knew the resident (99% reported always doing so) and the clinical strengths of the resident (96% reported always doing so). A specific statement on the overall strength of the recommendation was reported by 86% of participants, and 41% always included specific coded language on the strength of their recommendation. The LOR features that were most strongly associated with the participant's confidence level in writing an effective LOR included a description of the resident’s scholarly activity (p<0.0001), the resident’s ability to work as part of a team (p=0.02), and the LOR writer’s own background (p=0.03) (Table 3).

**Table 3:** Items included in LOR (% reporting always including a topic) stratified by confidence in LOR writing LOR: letter of recommendation

	Very confident	Somewhat confident	Not confident	p-value
Description of the training program	45%	54%	33%	0.05
Writer’s background	90%	86%	33%	0.03
Relationship with trainee	100%	100%	83%	1.0
Trainee clinical strengths	100%	91%	100%	1.0
Trainee scholarly activity	84%	60%	33%	<0.0001
Trainee communication style	52%	36%	33%	0.22
Trainee medical knowledge	81%	69%	50%	0.28
Trainee ability to work as part of a team	74%	82%	66%	0.02
Summary of trainee curriculum vitae	33%	14%	0%	0.22
Summary statement at end of LOR	87%	89%	66%	0.59
Specific code words at the end of LOR	48%	37%	17%	0.15

Participants reported variable use of first name and surname when referring to a resident, with 36% always referring to the applicant by professional address such as “Dr. Smith” or “Dr. Susan Smith,” 3% always referring to the resident by only their first name, and 61% using a combination. This was not associated with the academic rank of the LOR writer (p=0.30). Gender bias was always considered (with regard to word choice) when writing LOR by 28% of participants, whereas 35% reported never considering it. This did not vary across academic rank (p=0.70) or confidence level (p=0.23). There was no association between the consideration of gender bias and the use of first name versus professional address (p=0.71).

Interest in guidelines on best practices for LOR writing was reported by 87.1% of participants, regardless of their individual confidence level in writing LOR. There was no significant difference in the interest in receiving LOR writing guidelines between faculty who felt very confident compared to those with lower levels of confidence (92.8% vs. 83.9%, p=0.27).

## Discussion

Fellowship PDs assign a high value to LOR during the application review process; however, the lack of available guidelines for letter writers makes LOR writing an inconsistent process [1]. We found that >50% of faculty at our institution did not feel very confident that their LOR communicated the necessary information to fellowship PDs. Confidence is particularly low among faculty with lower academic rank who comprise the bulk of our participants. We also found that those with leadership positions in training programs did not report higher levels of confidence, despite their exposure to reviewing LOR during this very process. The reasons why PDs do not have higher confidence in writing LOR may be due to a lack of a well-agreed-upon and widely recognized approach to this activity.

Participants who felt very confident were also significantly more likely to include a description of the trainee’s scholarly activity. This is important given previous studies demonstrating that fellowship PDs held scholarly activity as a key feature in assessing applicants and were valuable inclusions in LOR [2]. Additionally, we demonstrate that confident LOR writers were more likely to provide a brief description of who they were in the LOR which provides important context on how to view the LOR which is a feature not previously described in the literature. The inclusion of these features in LOR writing guidelines is important as the writer's longitudinal experience with trainees can put the strength of a recommendation into a better context.

Confidence in writing an effective LOR does not necessarily mean that the LOR actually contains the necessary information to allow for an appropriate assessment of a pediatric subspecialty fellowship application. It is notable though that some faculty respondents in our study who feel very confident omit features such as scholarly activity or a summary statement (that have previously been cited by pediatric fellowship PDs as key points in applications) to convey the overall level of recommendation [2]. This provides additional data to support the need for guidelines on writing LOR. Further, academic rank does not necessarily correlate with how often a faculty member writes LOR especially for those faculty members heavily involved in medical education. Our data suggest there is a cohort of pediatric faculty with lower academic rank who work less frequently with trainees that should be prioritized for faculty development in LOR writing.

Our study also demonstrates that a relatively low number of faculty systematically consider gender bias when writing LOR which is consistent across academic ranks and levels of confidence. The medical literature has demonstrated variable rates of gender bias in LOR across specialties including urology, ophthalmology, gynecology, and vascular surgery [23-25]. More specifically in pediatrics, word bias has been demonstrated in both clinical evaluations of pediatric residents as well as in LOR written for pediatric fellowships [20,21]. Given that our data demonstrates one-third of faculty respondents never consider gender bias when writing LOR for pediatric residents applying to fellowship programs, it is imperative that LOR guidelines include education on how gender bias influences LOR and offer tools (such as using online scoring algorithms) to combat this bias [26]. This data, which raises concern about acknowledging implicit bias, suggests that although there may be a preferred target population for improving confidence, all faculty should receive some instruction.

Our study has limitations. The survey asked participants to reflect on their personal habits in LOR writing and thus included an element of recall bias. Although the response rate was relatively high at nearly 60%, voluntary participation may reflect a particular subset of the overall pediatric faculty (such as those who write LOR more often) and thus may not generalize to the full population of pediatrics faculty at our institution. Further, this survey was conducted at a single center, and the results may not generalize to faculty letter writers at other institutions (including those with different distributions of academic rank). Similarly, these data may not be generalizable to other medical specialties.

Our study describes a clear interest among all faculty in receiving guidelines or education on writing LOR for fellowship applicants which was true even for those with high degrees of confidence in their ability to write an effective LOR. Given this significant interest, departmental leadership should look to provide faculty development in writing LOR. It is notable that among LOR writers who were not confident, only 33% described a scholarly activity of the resident and only 66% provided a summary statement on the strength of the LOR. Future development of guidelines supported by groups such as the Associate of Pediatric Program Directors and the Council of Pediatric Subspecialties would be ideal to help standardize the process nationally. More importantly, national guidelines may improve the ability of fellowship PDs to accurately judge LOR due to more effective writing especially if they limit implicit bias.

## Conclusions

The information conveyed in LOR in the fellowship application process plays a strong role as fellowship programs evaluate applicants. However, the confidence of pediatric faculty in writing LOR could be improved. There may be value in developing standardized guidelines for writing LOR and faculty development (at both institutional and national levels) to assist faculty who are writing LOR for trainees pursuing subspecialty fellowship positions, especially in light of our findings that 35% of LOR writers never considered gender bias when writing LOR. These developmental offerings would ideally focus on topics to include in the LOR to optimally reflect the resident’s abilities and include tools to combat implicit bias.

## Appendices

Supplement: survey tool

In the last 5 years, how many letters of recommendation have you written for residents?

1)     None

2)     1-2

3)     >2

How confident do you feel that your letters of recommendation help the fellowship programs make informed decisions about the resident?

1)     Very confident

2)     Somewhat confident

3)     Not confident

Have you ever declined to write a letter of recommendation?

1)     Yes

2)     No

How do you refer to the resident in the letter?

1)     Always as Dr. ____

2)     Sometimes as Dr. ___ and sometimes by first name

3)     Always by first name

How often do you consider gender bias when you select words to use in a letter of recommendation?

1)     A lot

2)     Sometimes

3)     Not at all

Which of the following do you request from the resident when you are asked for a letter of recommendation (all scored always vs sometimes vs never)

A)     1-on-1 meeting to discuss

B)     Updated CV

C)     Draft of personal statement

D)     Draft of letter of recommendation for you to review/edit

E)     Specific topics to include in the letter

What components do you include in your letter? (Always vs Sometimes vs Never)

A)     Brief description of the resident’s current training program

B)     Brief statement of who you (the writer) are

C)     How do you know the resident

D)     Clinical strengths of the resident

E)     Scholarly activity of resident

F)      Communication style of the resident

G)     Medical knowledge of resident

H)     Ability of resident to work as part of a team

I)       Summary of resident’s CV

J)       Summary statement at the end of the letter to describe the strength of your overall recommendation

K)     Specific code phrases such as “absolute highest recommendation” or “top __% of residents that I’ve worked with”

If the department offered you a colleague to review letters you have written (to confirm they accurately portray your impression/recommendation), how likely are you to use that service?

1)     Likely

2)     Not likely

Are you interested in receiving recommendations on how to write effective letters of recommendation?

1)     Yes

2)     No

During the last 12 months, how often do you typically work with residents?

1)     More than once a week

2)     Once a week

3)     Less than weekly

Are you currently an MGfC residency or fellowship program director or assistant/associate program director?

1)     Yes

2)     No

What is your current Harvard Medical School rank?

1)     Professor

2)     Associate professor

3)     Assistant professor

4)     Instructor

5)     Other
